# Marked gut microbiota dysbiosis and increased imidazole propionate are associated with a NASH Göttingen Minipig model

**DOI:** 10.1186/s12866-022-02704-w

**Published:** 2022-12-01

**Authors:** Ditte Olsen Lützhøft, Tim Sinioja, Berit Ø. Christoffersen, Rasmus Riemer Jakobsen, Dawei Geng, Hajar Fauzan Bin Ahmad, Ellen Marie Straarup, Karen-Margrethe Pedersen, Witold Kot, Henrik Duelund Pedersen, Susanna Cirera, Tuulia Hyötyläinen, Dennis Sandris Nielsen, Axel Kornerup Hansen

**Affiliations:** 1 Department of Veterinary and Animal Sciences, Faculty of Health and Medical Sciences, University of Copenhagen, 1871 Frederiksberg C, Denmark; 2 School of Science and Technology, Örebro University, 702 81 Örebro, Sweden; 3 Global Drug Discovery, Novo Nordisk, Måløv, Denmark; 4 Department of Food Science, Faculty of Science, University of Copenhagen, 1958 Frederiksberg C, Denmark; 5 Present address: Novo Nordisk Foundation Center for Basic Metabolic Research, University of Copenhagen, 2200 Copenhagen, Denmark; 6 Present address: Faculty of Industrial Sciences & Technology, Universiti Malaysia Pahang, Lebuhraya Tun Razak, 26300 Gambang Kuantan, Pahang Malaysia; 7 Present address: Centre for Research in Advanced Tropical Bioscience (Biotropic Centre), Lebuhraya Tun Razak, 26300 Gambang Kuantan, Pahang Malaysia; 8 Department of Plant and Environmental Science, University of Copenhagen, 1871 Frederiksberg C, Denmark; 9 Ellegaard Göttingen Minipigs, Sorø Landevej, Dalmose, Denmark

**Keywords:** Hyperglucagonemia, SCFA, pH, colon microbiota, Bile acids, Imidazole propionate

## Abstract

**Background:**

Gut microbiota dysbiosis is associated with the development of non-alcoholic steatohepatitis (NASH) through modulation of gut barrier, inflammation, lipid metabolism, bile acid signaling and short-chain fatty acid production. The aim of this study was to describe the impact of a choline-deficient amino acid defined high fat diet (CDAHFD) on the gut microbiota in a male Göttingen Minipig model and on selected pathways implicated in the development of NASH.

**Results:**

Eight weeks of CDAHFD resulted in a significantly altered colon microbiota mainly driven by the bacterial families *Lachnospiraceae* and *Enterobacteriaceae*, being decreased and increased in relative abundance, respectively. Metabolomics analysis revealed that CDAHFD decreased colon content of short-chain fatty acid and increased colonic pH. In addition, serum levels of the microbially produced metabolite imidazole propionate were significantly elevated as a consequence of CDAHFD feeding. Hepatic gene expression analysis showed upregulation of mechanistic target of rapamycin (mTOR) and Ras Homolog, MTORC1 binding in addition to downregulation of insulin receptor substrate 1, insulin receptor substrate 2 and the glucagon receptor in CDAHFD fed minipigs. Further, the consequences of CDAHFD feeding were associated with increased levels of circulating cholesterol, bile acids, and glucagon but not total amino acids.

**Conclusions:**

Our results indicate imidazole propionate as a new potentially relevant factor in relation to NASH and discuss the possible implication of gut microbiota dysbiosis in the development of NASH. In addition, the study emphasizes the need for considering the gut microbiota and its products when developing translational animal models for NASH.

**Supplementary Information:**

The online version contains supplementary material available at 10.1186/s12866-022-02704-w.

## Background

Obesity [1] and type 2 diabetes [2] may lead to fatty liver and non-alcoholic steatohepatitis (NASH), which is characterized by hepatic inflammation, fibrosis, steatosis, hepatocyte ballooning and insulin resistance [2]. In addition, these metabolic disorders have also been linked to gut microbiota dysbiosis, characterized by low bacterial diversity, increased intestinal permeability followed by translocation of bacterial endotoxins and gut derived inflammatory mediators in addition to a shift in the gut microbial products absorbed into systemic circulation [3, 4].

Previously, changes in the total bile acid (TBA) and bile acid (BA) profile have been linked to gut microbiota dysbiosis and NASH [3]. The classic pathway of liver produced primary BAs from cholesterol is under the control of the enzyme Cholesterol 7a-hydroxylase (CYP7A1). When the primary BAs are produced they are transported to the small intestine through the bile ducts where they exert digestive functions, act as signaling molecules, and regulate multiple physiological functions [5]. Approximately 90% of the luminal conjugated primary BAs are actively reabsorbed in the terminal ileum [3]. The remaining luminal BAs are deconjugated by bile salt hydrolases (BSHs) followed by 7a-dehydroxylation into secondary BAs by 7a-dehydroxylase produced by the intestinal microbiota and hereafter they are passively reabsorbed and returned to the liver via the portal vein [3, 5]. In addition, gut microbial BA hydroxysteroid dehydrogenases (HDSHs) performs the reversible epimerization of primary BAs into different BA intermediates contributing to the complexity of the total bile acid equilibrium [6]. The BA bioconversions modulate the signaling properties of the BAs through G protein-coupled membrane receptor 5 (TGR5) and nuclear farnesoid X receptor (FXR), that in return can regulate host metabolic pathways [7]. Gut microbiota dysbiosis interrupts the TBA equilibrium through a changed capacity for conversion of toxic hydrophobic BAs to hydrophilic BAs, thereby reshaping the BA profile. The changed BA signaling cascades directly or indirectly regulates host metabolism through intestinal L-cell secretion of glucagon-like peptide 1 (GLP-1) alongside affecting pancreatic, hepatic and colonic gene expression of TGR5, FXR and CYP7A1 altering insulin secretion, hepatic regulation of gluconeogenesis and the synthesis of primary BAs [7].

Recently, the gut microbial product imidazole propionate has been linked to impaired insulin signaling in mice and humans [8–10]. Imidazole propionate is produced in the colon and causes decreased hepatic insulin signaling by activating mechanistic target of rapamycin complex 1 (mTORC1) and S6K1 phosphorylation which is leading to phosphorylation of insulin receptor substrate 1 (IRS1) and insulin receptor substrate 2 (IRS2) targeting them for degradation [8]. An essential link in mTORC1 activation is its translocation to the lysosome, which is mediated by rag guanosine triphosphatases (GTPase), and where it is activated by another GTPase, i.e. Ras homolog enriched in brain (or Ras Homolog, MTORC1 binding (RHEB)) [11]. Imidazole propionate is produced from histidine, a process that is performed by the bacterial- produced enzyme urocanate reductase (*urdA*) [8]. *UrdA* is active at neutral pH [8, 12]. Normally, the intestinal pH in the proximal colon is maintained at pH 6.1–6.5 by the production of short-chain fatty acids (SCFA) [13] and rises to pH 6.6–7 in the distal colon in humans and pigs [13, 14]. However, the pH balance may be disrupted by gut microbiota dysbiosis with decreased production of SCFA, creating an environment that facilitates the production of the imidazole propionate [15]. Additionally, SCFA are themselves involved in the regulation of host metabolism [16], immune functions [17] and the development of NASH/NAFLD [18].

The understanding of the gut microbiota dysbiosis as observed in NASH/NAFLD [19] on the gut-pancreas-liver axis [20] and newly established liver-a-cell axis, important for glucagon receptor (GCGR) signaling in humans [21, 22], are far from complete. This is partly due to the lack of adequately translational animal models. Choline deficiency in humans are associated with liver dysfunction and humans deprived of choline develops either fatty liver or cell death [23]. The choline deficient and methionine defined diet are used as it recapitulate the NASH phenotype seen with NASH patients as liver steatosis [23]. To further characterize a newly established minipig model of NASH [24], where castrated male Göttingen Minipigs were fed a choline-deficient, amino acid-defined high fat diet (CDAHFD) over the course of 8 weeks, the consequences of the diet for the gut microbiota, its products and the resulting effects on selected metabolic parameters were evaluated. In this study, we identified gut microbiota dysbiosis, increased imidazole propionate concentrations alongside changes in the TBA and BA profile as possible contributing factors to hyperglucagonemia and the development of NASH.

## Results

### CDAHFD fed minipigs display a shift in colon gut microbiota leading to decreased SCFA production and a change in pH

**Fig. 1:**
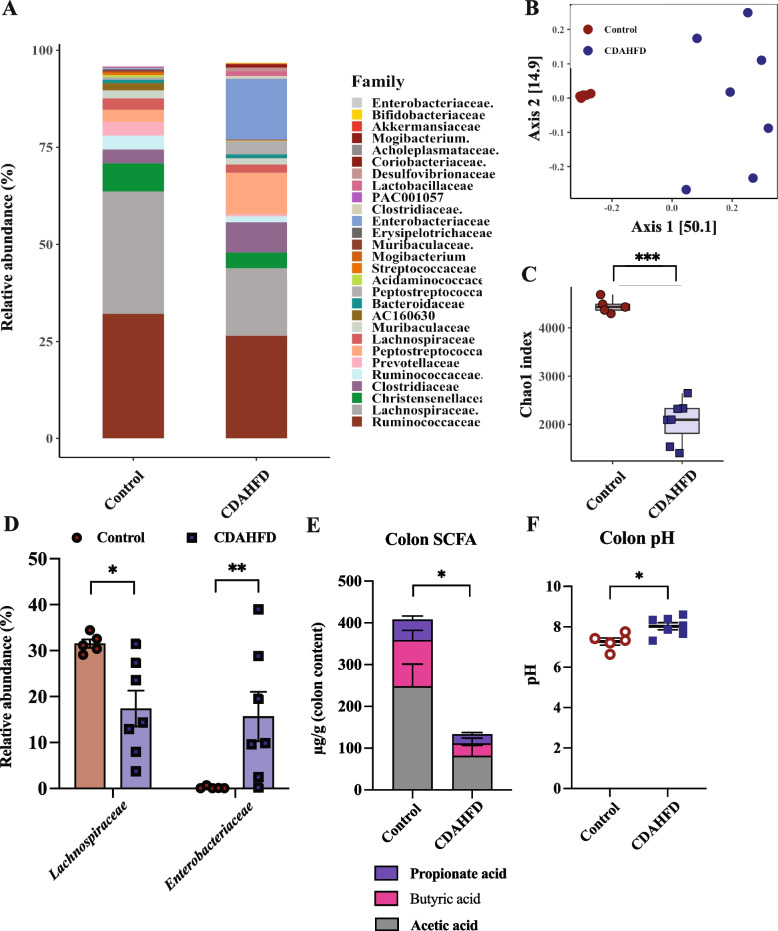
CDAHFD fed Göttingen Minipigs experienced a decreased amount of short-chain fatty acid resulting in the colon increasing the pH Colon microbiome composition of Göttingen Minipigs after 8 weeks of either control (*n* = 5) or choline-deficient amino acid defined high fat diet (CDAHFD) (*n* = 7) starting at age 8 weeks as determined by 16S rRNA amplicon gene sequencing: **A** Taxa summary **-** Bar plot illustrating the 28 most abundant bacterial families present as a consequence of differential feeing **B** PCoA plot using GUniFrac distance showing clear separation of the colon microbiota as a consequence of differential feeding; PERMANOVA, ANOSIM: R^2^ = 0.475, *p* = 0.0010, and **C** Boxplot showing Alpha diversity (Chao1 index) (25th percentile, 75th percentile, median, whiskers indicate max and min value; student’s t-test: *p* = 0.0000014); **D** Relative abundance (%) of bacterial families *Lachnospiraceae* and *Enterobacteriaceae;* **E** Metabolomics was used to determine the three most abundant short chain fatty acid (SCFA) butyric-, propionate- and acetic-acids (µg/g colon content) in the colon microbiota (mean + SEM); **F** pH measurement of colon content. Data are shown as individual values with mean ± SEM. *p < 0.05, **p < 0.01 and ***p < 0.001 mark the level of statistical significance

To evaluate the diet effect on colon microbiota of the minipigs we used 16S rRNA amplicon sequencing. Taxa-summary of families showed several major changes in bacterial composition dependent of diet (Fig. 1A, taxa summary for the individual minipigs supplementary FigS1). Supporting this, beta-diversity (generalized UniFrac) revealed a significant separation of colon microbiota between control and CDAHFD fed minipigs (ANOSIM: *R*^*2*^ = 0.48, *p* = 0.0010, Fig. 1B). Furthermore, CDAHFD reduced colon microbiota richness significantly compared to the control group (*p* = 0.0000014) (Fig. 1C). The main differences in bacterial relative abundances were observed for the *Lachnospiraceae* family and the *Enterobacteriaceae* family (*Escherichia* genus, supplementary Table S4) which were negatively and positively associated with CDAHFD, respectively (Fig. 1D).

Several bacterial families of minor relative abundance were found to compose a significantly larger proportion of the colon microbiota in the minipigs fed CDAHFD, these were the *Peptostreptococcaceae*, *Desulfovibrionaceae* and *Bacteroidaceae* families (supplementary Table S4). *Acholeplasmataceae*, *Streptococcaceae* and *Muribaculaceae* families, on the other hand, constituted a larger proportion of the colon microbiota in the control group (supplementary Table S4).

The three most common SCFAs were enumerated using GC-TOFMS from the colon contents. We found that these were significantly reduced, and especially butyric acid, with the minipigs fed CDAHFD compared to control (Fig. 1E). Because the consequence of a decreased SCFA content in the colon is a rise in pH we measured pH of the collected samples, finding that pH in the colon content of the minipigs fed CDAHFD was significantly increased compared to control (Fig. 1F, mean pH level at the colonic spiral junction: control pH 7.2 ± 0.18; CDAHFD pH 8.0 ± 0.17).

Taken together these data show a shift in colon microbiota, decreased SCFA content in colon and a rise in colonic pH following CDAHFD feeding.

### Circulating imidazole propionate and decreased hepatic insulin signaling associated with CDAHFD feeding

**Fig. 2:**
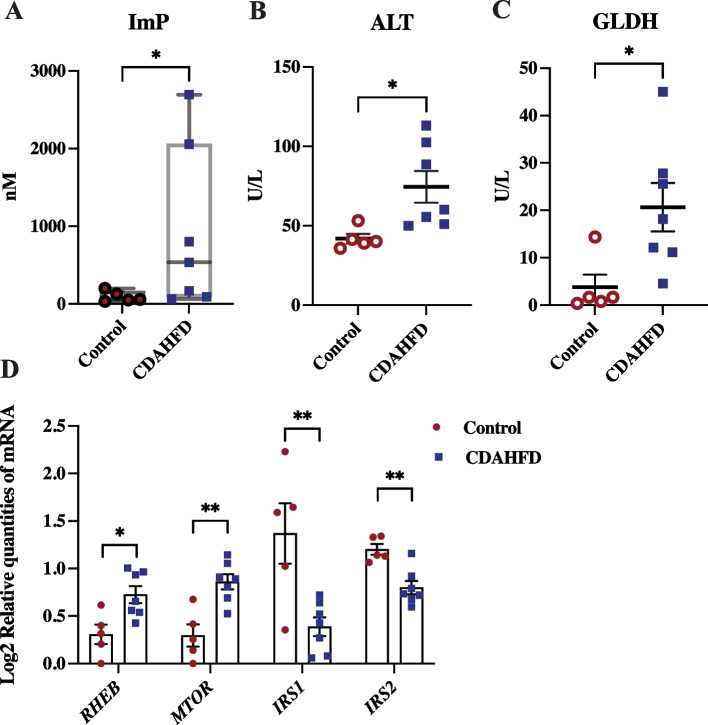
Imidazole propionate produced by the gut microbiota potentially impaired hepatic insulin signaling in a CDAHFD fed Göttingen Minipig Göttingen Minipigs were fed either a control (*n* = 5) or choline-deficient amino acid defined high fat diet (CDAHFD) (*n* = 7) for 8 weeks starting at age 8 weeks **A** Boxplot of Serum imidazole propionate (ImP) (nM) (25th percentile, 75th percentile, median, whiskers indicate max and min value); **B** Alanine transaminase (ALT) (U/L) (previously published in Pedersen and colleagues (2020) [24]) and **C** Glutamate dehydrogenase (GLDH) (U/L) (previously published in Pedersen and colleagues (2020) [24]); **D** Relative levels of hepatic mRNA (log2 relative quantities of mRNA) for Ras Homolog, MTORC1 binding (*RHEB*)*,* Mechanistic target of rapamycin (*MTOR*)*,* insulin receptor substrate 1 (*IRS1*) and insulin receptor substrate 2 (*IRS2*), fdr adjusted. Data are shown as individual values with mean ± SEM. *p < 0.05 and **p < 0.01 mark the level of statistical significance

An increased colonic pH has been reported to affect the bacterial metabolite production and the subsequent absorption into host circulation [25], so we examined this further in our study. The gut microbial metabolite imidazole propionate is produced by bacterial enzyme *urdA* [8]. Using LC-MS, we showed that the level of imidazole propionate in circulation of the minipigs fed CDAHFD was increased relative to the control group (Fig. 2A). Furthermore, we observed significantly elevated serum alanine aminotransferase (ALT) (Fig. 2B) and GLDH (Fig. 2C) levels, both biomarkers associated with hepatocyte apoptosis and mitochondrial damage [26], in minipigs fed CDAHFD. In the literature, ALT has been associated with decreased insulin sensitivity [27] and therefore we used qPCR to investigate genes in the mTORC1 complex and insulin receptor substrate. We observed that 8 weeks of feeding with CDAHFD significantly increased the expression of hepatic *RHEB* and *MTOR* genes (Fig. 2D), whereas hepatic expression of *IRS1* and *IRS2* were significantly decreased (Fig. 2D).

Taken together, the findings fit with increased imidazole propionate levels causing activation of mTORC1 and impaired hepatic insulin signaling in the CDAHFD fed minipigs.

### Connection between circulating imidazole propionate and hyperglucagonemia

**Fig. 3:**
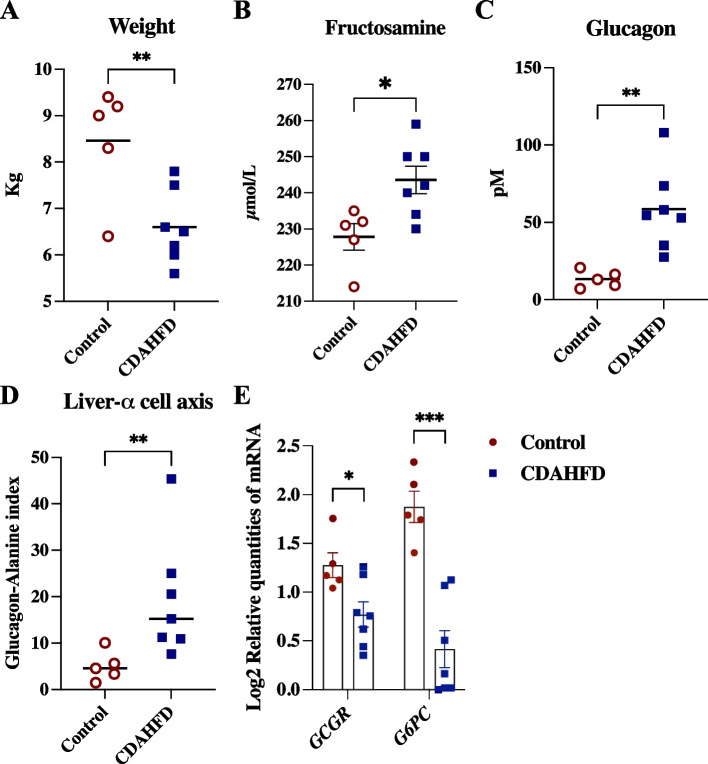
CDAHFD fed Göttingen minipigs experienced hyperglucagonemia and altered gene expression in the liver. Göttingen Minipigs were fed either a control (*n* = 5) or choline-deficient amino acid defined high fat diet (CDAHFD) (*n* = 7) for 8 weeks starting at age 8 weeks. **A** Body weight (Kg), **B** Fasting fructosamine (µmol/L) and **C** Fasting glucagon (pM) (previously published in Pedersen and colleagues (2020) [24]); **D** Liver-a-cell axis: Glucagon-alanine index (pM/µM); **E** Relative gene expression (log2 relative quantitives of mRNA) of hepatic glucagon receptor (*GCGR*) and glucose-6-phosphatase subunit (*G6PC*) determined by quantitative real-time PCR, fdr adjusted. Data are shown as individual values with mean ± SEM. *p < 0.05, **p < 0.01 and ***p < 0.001 mark the level of statistical significance

As previously published [24] metabolic parameters did not show a difference between CDAHFD fed and control fed minipigs in neither fasting insulin, glucose nor homeostatic model assessment of insulin resistance (HOMA-IR) (supplementary Fig. S2A-C). BW was significantly decreased while fructosamine was significantly increased with the CDAHFD fed minipigs compared to control (Fig. 3A-B). A significantly higher level of glucagon was observed in the CDAHFD group (Fig. 3). Supporting the hyperglucagonemic state, we found the glucagon-alanine index to be significantly higher in pigs fed CDAHFD (Fig. 3D). This significance was mainly driven by major differences in glucagon levels between diets as alanine levels did not deviate significantly (supplementary Fig. S2D). Likewise, the total serum level of measurable non-branched amino acids was unchanged between the groups (supplementary Fig. S2E). Furthermore, gene expression analysis revealed that hepatic gene expression of *GCGR* and *G6PC* were decreased in CDAHFD fed minipigs compared to control (Fig. 3E). Multiple linear regression was used to test if imidazole propionate and total serum amino acid could predict the glucagon level. Imidazole propionate was found to be a statistically significant predictor for the level of glucagon (*p* = 0.0068), while this was not the case for total amino acid (*p* = 0.16) (supplementary Table S5).

Taken together, these observations could connect the circulating level of imidazole propionate to the hyperglucagonemic state.

### Gut microbiota dysbiosis and serum TBA may have contributed to hepatic fibrosis severity

**Fig. 4:**
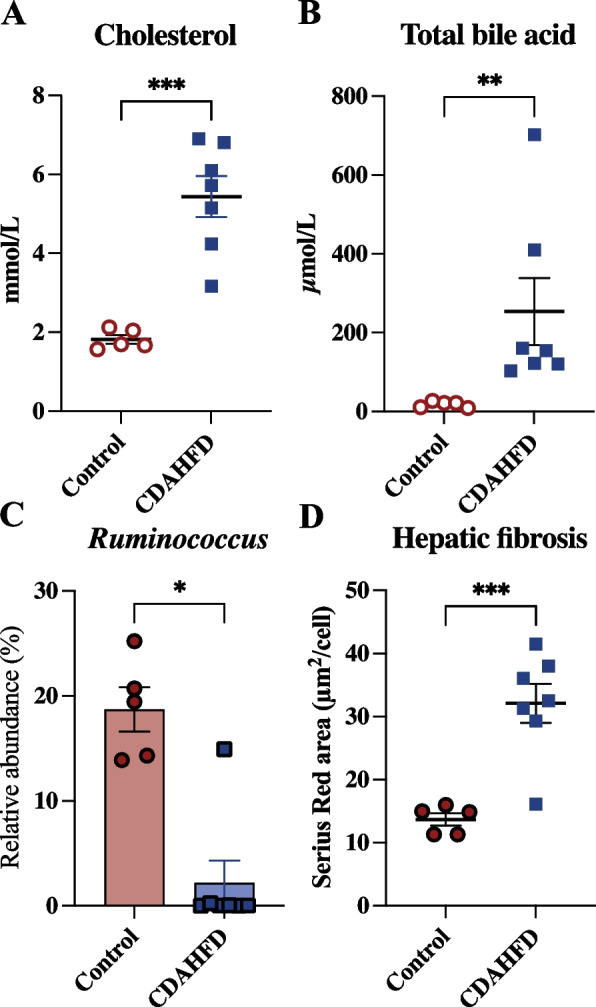
Gut microbiota dysbiosis and serum total bile acid may be related to hepatic fibrosis severity. Göttingen Minipigs were fed either a control (*n* = 5) or choline-deficient amino acid defined high fat diet (CDAHFD) (*n* = 7) for 8 weeks starting at age 8 weeks. **A** Cholesterol level (mmol/L) (previously published by Pedersen and colleagues (2020) [23]) and **B** Serum total bile acid (µmol/L); 16S rRNA amplicon sequencing was used to determine the composition of the colon microbiome and was found to contain **C** *Ruminococcus* genus (Relative abundance (%)) **D** Hepatic fibrosis level determined using histology pico Sirius Red image analysis (µm^2^/cell) (previously published by Pedersen and colleagues (2020) [24]). Data are shown as individual values with mean ± SEM. *p < 0.05, **p < 0.01 and ***p < 0.001 mark the level of statistical significance

We observed an increased level of circulating cholesterol and circulating TBA (Fig. 4A and B) as well as increased accumulation of hepatic cholesterol [24] in the CDAHFD fed minipigs compared to control. An increase in TBA alongside a change in BA profile have in the literature been linked to gut microbiota dysbiosis in NASH patients [28]. A changed BA profile could arise from a decreased conversion of toxic to non-toxic BA by HDSHs [6] and therefore we investigated this further. Members of the *Ruminococcus* genus, reported to contain HDSHs [6], were significantly decreased in the colon microbiota of the CDAHFD fed minipigs compared to control (Fig. 4C). A shift in the BA profile have been linked to the severity of hepatic fibrosis in NASH [3]. The minipigs fed CDAHFD had increased liver fibrosis compared to control (Fig. 4D). Multiple linear regression was carried out to predict hepatic fibrosis level, and the individual predictors, members of the *Ruminococcus* genus (*p* = 0.021) and TBA (*p* = 0.042) (supplementary table S6), were both significant predictors of hepatic fibrosis in this model. In addition, there was a collective significant effect of members of the *Ruminococcus* genus and TBA on the hepatic fibrosis level (*p* = 0.0083, *R*^*2*^ = 0.58) (supplementary Table S6). Collectively, this suggests that there could be a connection between the colon microbiome, the TBA profile and the severity of hepatic fibrosis in the CDAHFD fed minipigs.

## Discussion

The focus in the area of gut microbiota and NASH have mainly been on bacterial secretion of lipopolysaccharides and endotoxins, increased bacterial capacity for ethanol production and changed BA profile in addition to effects on the immune response [3]. In this study, we identified a shift in the colon microbiota alongside a decreased SCFA production, a rise in colonic pH and a changed BA profile in CDAHFD fed Göttingen Minipigs, a recently established animal model of NASH. We hypothesized that this resulted in the bacterial enzymatic production of imidazole propionate in the colon lumen, and that this gut microbial product could have a role in the pathogenesis of NASH. The study included low group size which must be taken into account when interpreting the data.

The colon microbiota of the CDAHFD fed minipigs had a significantly lower richness compared to control fed minipigs and there was a significant clustering of colon microbiota with diet. The butyrate producing members of *Lachnospiraceae* [29] as well as the *Muribaculaceae* family, who are propionate producers [30] (supplementary Table S4), were decreased in relative abundance and may be the cause of the observed decreased capacity for SCFA production. Meanwhile, the *Enterobacteriaceae* family (*Proteobacteria* phylum, supplementary Fig. S3A), mainly attributed to the *Escherichia* genus (supplementary Table S4), was increased in abundance in response to CDAHFD. This inverse relationship in relative abundance between the *Lachnospiraceae* and *Enterobacteriaceae* families has previously been observed in the gut microbiota of NASH patients [3, 31]. The pH of the healthy colon is slightly acidic in pigs and humans (pH 6.1–6.4 in the proximal colon rising to 6.7–7 in the distal colon [13, 14]) and is mainly maintained by SCFA production [13]. When colonic SCFA production decreases, the pH in that area increases [25], as also observed in this minipig study in addition to a significant negative correlation between the two (r = - 0.74, *p* = 0.006, supplementary Fig. S3B). This pH change may be associated with the increased growth of the *Enterobacteriaceae* family observed in the colon microbiota with the CDAHFD fed minipig, as this bacterial family may have favorable growth potential when the pH increases in the colon lumen [32]. However, it must be noted that the pH measured in this study was in the colon spiral junction, which present a limitation for interpreting the pH in the proximal colon. Nonetheless, pH profiles obtained from pig indicate that the proximal colon pH is lower compared to distal colon pH [14]. Furthermore, when the colon microbiome contains *urdA*-producing bacteria and proximal colon pH rises to neutral, the bacterial enzyme *urdA* is active and facilitates the production of imidazole propionate from histidine [8]. The microbial capacity for imidazole propionate production in the present study did not depend on the presence of histidine, as it was equal for the two diets (supplementary Table S1), but probably rather on the concentration and activity of *urdA* [9]. After production, imidazole propionate is transported through the portal vein from the gut to the liver [33], where it induces decreased hepatic insulin signaling through mTORC1 complex and S6K1 phosphorylation by blocking IRS1 tyrosine 612 phosphorylation and inhibiting insulin Akt phosphorylation [8]. We speculate that a consequence of CDAHFD is hepatic activation of mTORC1 and decreased expression of *IRS1* and *IRS2* as also indicated by gene expression analysis. Lastly, decreased hepatic autophagy mediated by mTORC1 causes acute liver injury [34] and is therefore already a described “player” in the pathogenesis of NASH.

In minipigs fed CDAHFD, we observed fasting hyperglucagonemia, but not hyperaminoacidemia. In addition, the CDAHFD fed minipigs had a significantly higher serum glucagon-alanine index, exclusively related to the glucagon level, and a decreased gene expression of hepatic *GCGR* and *G6PC* compared to control group. This indicates impaired regulation of hepatic gluconeogenesis, as glucagon, in theory, stimulates gluconeogenesis and an increased gene expression level of hepatic *GCGR* and *G6PC* would be expected in such a scenario [35]. Furthermore, histology confirmed pronounced hepatic steatosis with the CDAHFD fed minipigs [24] which is reported to cause reduced hepatic glucagon sensitivity in rats [36]. Collectively, this suggests that CDAHFD fed minipigs experience impaired hepatic glucagon receptor signaling. Liver dysfunction in NAFLD patients has been linked to hypersecretion of glucagon independently of a change in glucose tolerance [37] and is instead being attributed to a disruption of the liver-a-cell axis [21, 22]. Impaired hepatic glucagon signaling leads to decreased ureagenesis followed by hyperaminoacidemia and compensatory secretion of glucagon by a-cells [38, 39]. In addition, Solloway and colleagues (2015) [38] describes how activation of mTOR in a-cells by amino acids, measured through ribosomal protein S6, can cause a-cell hyperplasia. Liver amino acid catabolism is blocked as a consequence of impaired glucagon signaling in the liver which increases the circulating level of amino acids [38]. As we did not observe hyperaminoacidemia in the minipigs it can be speculated that mTORC1 activation can also be induced by imidazole propionate, not only amino acids, in a-cells and cause a-cell hyperplasia resulting in glucagon hypersecretion. However, this must be addressed in a future investigation that includes pancreas histology to substantiate this. The CDAHFD fed minipigs had a significantly lower weight compared to the control group at euthanasia. This was linked to an early flavor dislike from this group of minipigs. The flavor was adjusted, and the weight development of the diet groups were similar (data not shown) but the CDAHFD minipigs were unable reach the weight of the control group in the remaining time of this study. A later study confirmed that the same CDAHFD significantly increased the weight of Göttingen Minipigs compared to control [40]. Of note, the difference in feeding pattern in CDAHFD fed minipigs may have resulted in a difference in the colon microbiome and microbial products in circulation and is therefore considered as a limitation of the study. Obesity is proposed to be a main driver of hyperaminoacidemia [22], and since the CDAHFD fed group was not characterized as obese this could explain the non-existing hyperaminoacidemia state in the CDAHFD minipigs. HOMA-IR was similar in the diet groups and a correlation between glucagon-alanine index and HOMA-IR (r = - 0.065, *p* = 0.85, supplementary Fig. S3C), as described in Albrechtson and colleagues (2018) [21, 22], was not observed. However, Albrechtson and colleagues (2018) [21] describes that approximately 16% of the variation in serum glucagon can be explained by amino acids and HOMA-IR which leaves the possibility for a proportion of the variance to be explained by insulin resistant a-cells [41] or gut microbial products in circulation as proposed here. Of note, this study did not include a glucose challenge or postprandial parameters, which mechanistically is different from the fasted state, preventing interpretation of events in the peripheral tissue otherwise important for conceiving an extended metabolic “picture” for this model.

The level of circulating cholesterol as well as accumulated hepatic cholesterol were accompanied by a significantly increased level of circulating TBA in the minipigs fed CDAHFD. This suggests that the production of primary BA was increased in these minipigs. However, as the CDAHFD contained the primary BA cholic acid (supplementary Table S1) this is contributing to the increased level of TBA. In the classic pathway for production of primary BA the rate limiting enzyme is CYP7A1. The expression of *CYP7A1* has in literature been described to be regulated by glucagon in human primary hepatocytes [42]. Circulating glucagon, through the glucagon receptor, induces cAMP that acts as a second messenger to activate protein kinase A (PKA). The resulting signaling pathway of activated PKA represses CYP7A1 promoter activity and consequently the expression of the gene [42]. We observed a significant positive association between serum glucagon and TBA (r = 0.91, *p* < 0.0001, supplementary Fig. S3D), and therefore, it can be speculated that the decreased expression of *GCGK* may result in decreased activation of PKA allowing *CYP7A1* overexpressing and consequently continuously hepatic production of primary BAs. This potentially conflicts with the well-established theory of BA activation of TGR5 which stimulates the intestinal L-cells to produce GLP-1 and favors pancreatic insulin secretion which suppress glucagon secretion [7]. However, this is believed to be a postprandial response in contrast to the fasted state evaluated in this study. Additionally, we observed that CDAHFD induced an increase in the abundance of bacteria with a known ability to 7a-dehydroxylate deconjugated primary BAs (genera *Clostridium* and *Eubacterium*; supplementary Fig. S3E) [5] potentially leading to a higher production of secondary BAs compared to the control minipigs. Different genera belonging to the *Ruminococcaceae* family were upregulated in either control or CDAHFD fed minipigs (supplementary Table S4), but species belonging to the *Ruminococcus* genus were significantly decreased in the CDAHFD minipigs. Lee and colleagues (2020) [43] showed that the *Ruminococcaceae* family had an inverse relationship with fibrosis severity in non-obese NASH patients. In addition, they showed that colonizing CDAHFD C57BL/6 J mice (NASH-induced) with *Ruminococcus faecis* alleviated liver damage [43]. In line with this, a multiple linear regression model predicted a positive effect of the *Ruminococcus* genus on fibrosis level in the liver of minipigs examined in this study, suggesting that members of the *Ruminococcus* genus could be a contributing factor in alleviation of hepatic fibrosis also in this species. In contradiction to this, Boursier and colleagues (2016) [4] reported that the *Ruminococcus* genus, although comprising a smaller part of the faecal microbiota in NASH patients, was positively associated with the severity of fibrosis on a significant level. NASH patients experience a dysregulation of BAs resulting in an elevated circulating level of TBA alongside a changed BA profile [28]. *Ruminococcus* generates HSDHs converting toxic BAs into ursodeoxycholic acid making them more water-soluble and less toxic to human cells [6]. Likewise, BA related pathways “glycine, serine, and threonine metabolism” and “taurine and hypotaurine metabolism” were previously identified as being elevated in faecal samples from NASH patients [28]. In this study, we observed a rise in serum threonine (supplementary Fig. S3F). Taken together, this indicates a shift in the BA profile as a consequence of CDAHFD feeding. Abnormally high levels of BAs as well as accumulation of potential toxic BAs are reported to damage the bile duct epithelium elevating the biliary pressure causing a rupture and exposing the hepatocytes to a high concentration of BAs, infiltration of inflammatory cells ending in apoptosis or necrosis [44] ultimately contributing to formation of hepatic fibrosis.

## Conclusion

**Fig. 5:**
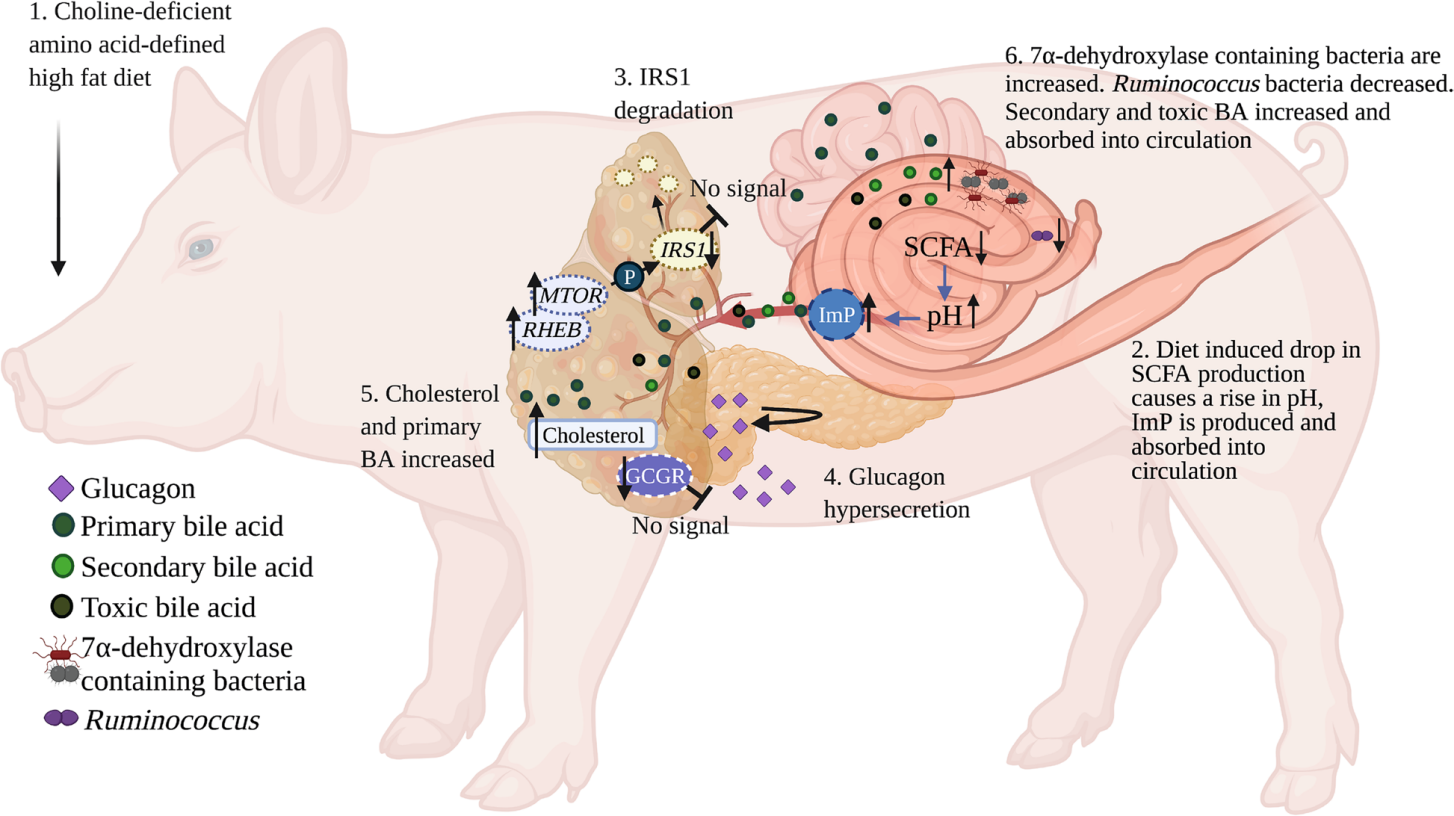
Proposed mechanism for imidazole propionate and bile acids in a CDAHFD fed Göttingen Minipig. CDAHFD indued change in the colon microbiota decrease SCFA production and increase the colonic pH. The increased pH activates bacterial produced enzymen *urdA* facilitating the production of imidazole propionate (ImP) which is subsequently absorbed into circulation with the minipig. Hereafter it reaches the liver and activates mTORC1 protein complex that in return starts a phosphorylation cascade resulting in degradation of *IRS1* resulting in impaired hepatic insulin signalling*.* We suggest that glucagon hypersecretion potentially caused by imidazole propionate activation of mTOR in pancreatic a-cells causes a-cell hyperplasia resulting in secondary hyperglucagonemia. Furthermore, CDAHFD increased circulating and hepatic cholesterol alongside an increased total bile acid. This likely results in an increased production of primary bile acids (BAs) which are absorbed into the small intestine. The majority of the primary BAs will be reabsorbed into in distal small intestines. However, a proportion reaches the colon and are converted to secondary BAs by 7a-dehydroxylase containing bacteria. In addition to these events, there is a decrease in the abundance of species belonging to the *Ruminococcus* genus possibly resulting in a lower conversion of toxic bile acid. This changes the BA profile and may contribute to the severity of hepatic fibrosis in this NASH minipig model. Created with Biorender.com and appropriate copyright permission have been obtained (agreement number: ZV24LQR2TQ)

In summary, 8 weeks of CDAHFD feeding to Göttingen Minipigs, an animal model displaying some of the hallmarks of human NASH, induced a shift in the colon microbiota, a decreased colon SCFA production in addition to a rise in colon luminal pH. This shift in the colon microbiota favored microbial production of imidazole propionate, which was associated with impaired hepatic insulin signaling, hyperglucagonemia, decreased expression of the glucagon receptor and disruption of the liver-a-cell axis. In addition, the TBA level was increased with indications of a changed profile potentially playing a role in the formation of the increased hepatic fibrosis observed in this model (Fig. 5). Results from the present study underlines the potential importance of the gut microbiota and gut microbial products as contributors to metabolic dysregulation and the NASH pathogenesis in Göttingen Minipigs, and many of the associations observed here seem to translate well to observations in humans.

## Methods

### Ethical statement

The animal study was carried out in accordance with the EU directive 2010/63/EU and the Danish Animal Experimentation Act (LBK 1306 from 23/11/2007 with 2011 amendments), and was approved by the Animal Experiments Inspectorate, Ministry of Food, Denmark (license number: 2016-15-0201- 01078).

### Göttingen Minipigs diet study

Twelve male Göttingen Minipigs (Ellegaard Göttingen Minipigs, Dalmose, Denmark) were castrated at 7 weeks of age and thereafter fed with either control diet (Minipig Expanded, Special Diets Service, Witham, United Kingdom) (*n* = 5), choline-deficient amino acid defined high fat diet (CDAHFD, Special Diets Service, Witham, United Kingdom) containing 1% cholesterol, 0.35% cholic acid, 30% fat (cocoa butter and milk fat), 0.1% methionine, no choline, and either 20% fructose or sucrose (CDAHFD-F *n* = 3, CDAHFD-S *n* = 4) (diet specification: supplementary Table S1). These diets were given for 8 weeks. The CDAHFD diets had the same energy content, and the sugar type did not significant influence the findings in the CDAHFD groups or the development of NASH [24], also suggested in the literature [45], therefore they were analyzed as one group: CDAHFD (*n* = 7). The pigs had ad libitum access to fresh drinking water and were fed twice daily with an access to food for 1 hour. The control group was fed according to internal standards (3–4% of their body weight) where the CDAHFD group was offered 75% of this amount of food (weight basis), i.e. an isocaloric amount relative to control [24]. The CDAHFD group had decreased appetite in the beginning. However, this was corrected over a period of 3–4 weeks by adding different flavors and ending with banana cream flavor. The minipigs were group-housed according to their respective diets and had straw as with a room temperature maintained at 22–24 °C and lights were on between 6 a.m. and 7 p.m. The minipigs had access to a heating lamp and their body weights were monitored. At euthanasia the pigs were fasted overnight and deeply anesthetized using intramuscular injection of a mixture containing 0.25 mg/kg of butorphanol and 1.25 mg/kg of each of the following: tiletamine, zolazepam, ketamine, and xylazine before being exsanguinated. For detailed description of the NASH phenotype of these minipigs see Pedersen and colleagues (2020) [24].

### Tissue and faecal sampling

Colon samples were collected at euthanasia from the colon spiral junction of the minipigs using tools that were sterilized between each animal by the use of 70% ethanol. Tissue samples from the liver were collected and snap frozen on dry ice before being transferred to - 80 °C for later processing. For further details see Pedersen and colleagues (2020) [24].

### Histology

Liver fibrosis was determined as part of the phenotyping previously published by Pedersen and colleagues (2020) [24], using quantitative image analysis on picrosirius red stained liver sections.

### Biochemical measurements

At end study, as part of the phenotyping previously published by Pedersen and colleagues (2020) [24], a hematological analysis was on ethylenediaminetetraacetic acid (EDTA) full blood (Advia 2120i Hematology System, Siemens, Ballerup, Denmark) and a clinical chemistry analysis was performed on plasma (Advia 1800 Chemistry System). In EDTA plasma glucose, fructosamine, alanine aminotransferase (ALT) and glutamate dehydrogenase (GLDH) were measured, in addition to serum BA on a Cobas 6000® autoanalyzer (Roche Diagnos- tics GmbH, Mannheim, Germany). Insulin and glucagon were determined by Luminescent Oxygen Channeling Immunoassay as described by Pedersen and colleagues (2020) [24] using GLU 1F120 mAb conjugated acceptor beads and biotinylated GLU 2F7 mAb for glucagon.

### Serum and faecal metabolite analysis

All serum and faecal samples were randomized prior to the sample preparation. Aliquots of 30 µL blood serum were subjected for protein precipitation using 400 µL methanol, containing internal standards (Sigma-Aldrich, Germany). After vortex mixing, incubating on ice for 30 min and centrifugation at 9400 x g for 3 min, 350 µL of supernatants were collected. The supernatants were then evaporated under gentle nitrogen flow to dryness after which a two-step derivatization was performed. First, 25 µL of 20 mg/mL MOX reagent in pyridine was added and samples were incubated at 45 °C for 60 minutes. Secondly, 25 µL MSTFA was added, and samples were incubated once again for 60 min at 45 °C. Retention index mixture (10 µg/mL n-alkanes) was added to each sample before the analysis on Agilent 7890B gas chromatograph (GC) coupled to 7200 triple quad time of flight mass spectrometer (Q-TOF/MS) instrument. Initial helium flow was set to 1.2 mL/min, increasing to 2.4 mL/min after 16 minutes. Oven temperature program was kept at 50 °C for 5 minutes, with 20 °C/min increase to 270 °C, and then 40 °C/min to final temperature 300 °C (5 min). Samples with injection volume of 1 µL and 100:1 split ratio were injected using PTV injector set to 70 °C, heated to 300 °C at 120 °C/min. Zebron ZB-SemiVolatiles column (20 m length, 0.18 mm inner diameter, 0.18 µm film thickness) (Phenomenex Inc., USA) was used to achieve a chromatographic separation. Electron ionization (EI) source was set to 250 °C, 70 eV and 35 µA emission with 3 minutes solvent delay. Quadrupole was kept at 150 °C having 1.5 mL/min N_2_ collision gas flow. The data was acquired at 55–650 amu mass range and 200 ms/spectrum acquisition time. Six-points calibration curves at 0.1–80 µg/mL range contained compounds of interest from Sigma-Aldrich, Germany.

SCFAs extraction in faecal material was processed by adding 1 mL of 5 mM aqueous NaOH containing internal standard to 100 mg aliquots. Sample was homogenized with a micro pestle and mixed for 10 min at a shaker (300 rpm). After shaking, the sample was centrifuged for 20 min at 13200 x g at 4 °C. 300 µl of MQ water, 500 µL propanol/pyridine mixture solvent (v/v = 3:2), 100 µL of propyl-chloroformate were added to 500 µL of faecal water obtained after centrifugation. The sample was vortexed and ultrasonicated for 1 min. After adding 300 µL of hexane, the sample was vortexed and centrifuged for 5 min at 2000 x g. 300 µL from the hexane layer was collected in a glass vial. 200 µL of retention index standards in hexane were added before analysis. Another aliquot of 100 mg faecal sample for dry weights determination were freeze-dried overnight at - 50 °C. Acquisition of BCFAs was done using an Agilent 7890A GC to an Agilent 5975C MS equipped with an electron ionization (EI) source (230 °C). GC separation was achieved using a DB-5MS capillary column, 30 m × 0.25 mm i.d. × 0.25 µm film thickness (Agilent Technologies, Atlanta, GA, USA). The oven temperature was as follows: 45 °C (4 min); 10 °C/min to 70 °C; 3 °C/min to 85 °C; 5 °C/min to 110 °C; 30 °C/min to 300 °C (5 min). 1 µL of samples were injected in splitl mode (split ratio 5:1) and carried out by carrier gas (helium) at 260 °C with a constant flow of 1.0 mL/min. The data was acquired in scan mode and mass range was between 50 and 300 amu (additional details is supplied in supplementary section).

### Imidazole propionate analysis

The same protocol as in Koh and colleagues (2018) [8] was used was used to determine imidazole propionate plasma concentrations. In brief, imidazole propionate was quantified using ultra-performance liquid chromatography coupled to tandem mass spectrometry and serum samples were.

extracted using 3 volumes of ice-cold ice-cold acetonitrile in 1.5 ml polypropylene also containing internal standards. Derivatization to 5% hydrochloric acid in butanol was performed, samples were injected onto a C18 column (2.1 × 100 mm with 1.7 mm particles; Waters, Milford, MA) and separated using a gradient consisting of water with 0.1% formic acid and acetonitrile with 0.1% formic acid [8].

### Gene expression

50 mg of snap-frozen liver tissue were used for RNA isolation as described in [23]. Concentration, purity and integrity of the RNA samples were analyzed as described in [23]. All samples had good concentration, acceptable purity and a RQI (RNA quality index) between 8.9–10 and were all accepted for further processing.

Subsequently, cDNA synthesis was done in duplicate for each RNA sample as described in [23]. Two negative controls (without reverse transcriptase) were processed in parallel. The cDNA samples were diluted 16 times prior to quantitative real-time PCR (qPCR) and stored at - 80 °C.

Profiled genes were chosen based on imidazole propionate and GCGR signaling in the liver [6]: RHEB, Mechanistic Target Of Rapamycin Kinase (MTOR), IRS1, IRS2, Glucagon Receptor (GCGR) and Gluconeogenic Enzyme Glucose-6-phosphatase (G6PC). Primers were designed using Primer 3 or primer-Blast (gene sequences: supplementary Table S2).

QPCR was performed in a CFX96™ Real-Time System (Bio-Rad) using SsoAdvanced Universal SYBR® green supermix (Bio-Rad) following manufacturer’s recommendations. PCR cycling condition were: 30 seconds 95 °C followed by 40 cycles of 10 seconds denaturation at 95 °C and 30 seconds 60 °C annealing/extension ending with a melting curve (for more detailed description of this section see supplementary section).

### Measurement of pH

Measurement of pH in minipig colon samples was performed by homogenizing the samples in sterilized water in the ratio 1:3 and following using a calibrated pH meter (Consort multi-parameter analyzer C3041, Turnhout, Belgium).

### DNA extraction, sequencing and pre-processing of raw data

Library Preparation and Sequencing: The bacterial community composition was determined by Illumina NextSeq-based high-throughput sequencing (HTS) of the 16S rRNA gene V3-region, according to Krych and colleagues*.* (2018) [46] and is described in detail in the supplementary materials and methods. Briefly, the amplified fragments with adapters and tags were purified and normalized using custom made beads, pooled, and subjected to 150 bp pair-ended Illumina NextSeq (V3 region 16S rRNA) sequencing. The raw dataset containing pair-end reads with corresponding quality scores were merged and trimmed, followed by de-replication, purging of chimeric reads, and constructing high quality (97% similarity level) Operational Toxonomic Unit (OTU) that and taxonomically assigned using sintax coupled to the EZtaxon 16S rRNA gene reference database. The sequencing dept. was on average 68,194 read per sample before filtering going down to 62,426 after filtering.

Sequencing data pre-processing: The dataset was purged for OTU’s which were detected below 0.005% across all samples, and normalization was accomplished using MetagenomeSeq v 1.32.0 based on Cumulative Sum Scaling algorithm [47].

### Statistics, calculations and bioinformatic analysis

In general, to evaluate the difference between the two groups, students t-test or Mann-Whitney test were used and a *p*-value< 0.05 was considered statistically significant. The data set was evaluated for normality using QQ plots and Shapiro-Wilk test. The analysis was conducted in PRISM v.9.0 (GraphPad Software, San Diego, California USA, www.graphpad.com). Stepwise backwards regression with linear regression model (based on Akaike’s Informative Criterion) was used to predict glucagon level (dependent variable: glucagon, independent variable: Imidazole propionate and total amino acid level) and hepatic fibrosis level (dependent variable: hepatic fibrosis, independent variables: *Ruminococcus* genus and total bile acids, random = group). Fligner-Killeen’s test was used to test for homogeneity of variance. The analysis was conducted in R open-source statistical software v4.0.3.

Total serum non-branched chain amino acid concentration included measurable amino acids alanine, proline, glycine, serine, threonine, and aspartic acid (values below limit of detection (LOD) were set to LOD/2) and all other non-branched chain amino acid were below detection limit and set to LOD/2 (supplementary Table S3). For calculation of glucagon-alanine index the following formula was used: glucagon-alanine index = fasting serum glucagon [pmol/L] * fasting serum alanine [µmol/L] [21]. Raw data from gene expression analysis was processed in Genex 6 (MultiD Analyses AB, Gothenburg, Sweden), log2 transformed data was extracted and analyzed using Student’s t-test including fdr adjustment for multiple testing using Benjamin, Krieger, and Yekutieli in PRISM v9.0.1. For conducting bioinformatic analysis R open-source statistical software v4.0.3 using packages Vegan v2.5.7 [48], PhyloSeq v1.34.0 [49], MetagenomeSeq v1.32.0 [47], GUniFrac v1.1 [50], and DAtest v2.7.17 [51] were used. Bacterial differences between groups were detected using DAtest package (lli function) and Mann-Whitney. Differential clustering was evaluated using ANOSIM. For visualization of data PRISM v9.0.1 and GGplot2 v3.3.3 [52] were used.

## Supplementary Information

**Additional file 1.**

**Additional file 2.**

**Additional file 3.**

## Data Availability

The datasets generated and analysed during the current study are available at Open Science Framework (https://osf.io/7u5qw/), DOI 10.17605/OSF.IO/7U5QW. Sequences are available at the European Nucleotide Archive (ENA) with accession number PRJEB54561, http://www.ebi.ac.uk/ena/browser/view/PRJEB54561 .

## Abbreviations

ALT: Alanine Aminotransferase
BA: Bile Acid
BSHs: Bile Salt Hydrolase
CDAHFD: Choline-Deficient Amino Acid Defined High Fat Diet
CYP7A1: Cholesterol 7a-hydroxylase
EI: Electron Ionization
FXR: Nuclear Farnesoid X Receptor
GC: Gas Chromatograph
GCGR: Glucagon Receptor
GLDH: Glutamate Dehydrogenase
GLP-1: Glucagon-Like Peptide 1
GTPase: Rag Guanosine Triphosphatases
G6PC: Gluconeogenic Enzyme
HDSHs: Hydroxysteroid Dehydrogenase
HTS: High-Throughput Sequencing
IRS1: Insulin Receptor Substrate 1
IRS2: Insulin Receptor Substrate 2
LC-MS: Liquid Chromatography Mass Spectrometer
LOD: Limit of Detection; Glucose-6-Phosphatase
mTORC1: mechanistic Target of Rapamycin Complex 1
mTOR: mechanistic Target of Rapamycin
NAFLD: Non-Alcoholic Fatty Liver Disease
NASH: Non-Alcoholic Steatohepatitis
OUT: Operational Toxonomic Unit
PERMANOVA: Permutational Analysis of Variance
PKA: Protein Kinase A
qPCR: quantitative real-time PCR
RHEB: Ras Homolog Enriched in Brain, Ras Homolog, mTORC1 binding
SCFA: Short-Chain Fatty Acid
TBA: Total Bile Acid
TGR5: G Protein-Coupled Membrane Receptor 5
Q-TOF/MS: Quad Time of Flight Mass Spectrometer
