# Supplementary material for: Marked gut microbiota dysbiosis and increased imidazole propionate are associated with a NASH Göttingen Minipig model
Source: BMC Microbiol. 2022 Dec 1;22:287. doi: 10.1186/s12866-022-02704-w (PMC9717514; doi:10.1186/s12866-022-02704-w)
Supplement: Supplementary file 1 — Additional file 1. [file 12866_2022_2704_MOESM1_ESM.docx]

**Supplementary figures**

**Supplementary figure S1.** Colon microbiome composition of Göttingen Minipigs after 8 weeks of either control diet or choline-deficient amino acid defined high fat diet (CDAHFD) starting at age 8 weeks as determined by 16S rRNA amplicon gene sequencing. Taxa summary **-** Bar plot illustrating the 28 most abundant bacterial families present in the individual minipigs.

**Supplementary figure S2.** Göttingen Minipigs after 8 weeks of either control (n=5) or choline-deficient amino acid defined high fat diet (CDAHFD) (n=7) starting at age 8 weeks. **A.** Fasting serum insulin (pM) and **B.** Fasting glucose (pM) (previously publised by Pedersen et al. (2020) (23)); **C.** Homeostatic model assessment of insulin resistance (HOMA-IR): Insulin (nM)*Glucose (nM) and **D.** Bar plot of serum alanine (µg/mL) and **E. S**erum Total amino acid (AA) (µg/mL) concentration measured by gas chromatograph coupled to time of flight mass spectrometer. Data are shown as indivudual values with mean±SEM.

**Supplementary figure S3.** 16S rRNA gene tag-encoded pyrosequenced sequencing proximal colon microbiome from Göttingen Minipigs after 8 weeks of either control (n=5) or choline-deficient amino acid defined high fat diet (CDAHFD) (n=7) starting at age 8 weeks. **A.** Relative abundance (%) (mean+SEM ) of bacterial phyla Proteobacteria*,* DAtest statistics using DA.lli function on 16S rRNA amplicon sequencing data, fdr adjusted; Pearson correlation between **B.** SCFA: acetic acid, propionic acid and butyric acid (µg/g faecal content) and pH, **C.** Glucagon-alanine index and Homeostatic model assessment of insulin resistance (HOMA-IR; Insulin (nM)*Glucose (nM) and **D.** Serum glucagon (pM) and serum total bile acid (µmol/L). p<0.05 was considered significant and scatterplots show values on a log scale; **E.** 16S rRNA amplicon sequencing was used to determine bacteria that have been reported to contain 7α-dehydroxylase: *Clostridium* and *Eubacterium* families; and **F.** Serum threonine (µg/mL) measured by measured by GC-TOF/MS. *p<0.05 and **p<0.01 mark the level of statistical significance. Data are shown as individual values with mean±SEM.
