# Supplementary material for: Marked gut microbiota dysbiosis and increased imidazole propionate are associated with a NASH Göttingen Minipig model
Source: BMC Microbiol. 2022 Dec 1;22:287. doi: 10.1186/s12866-022-02704-w (PMC9717514; doi:10.1186/s12866-022-02704-w)
Supplement: Supplementary file 2 — Additional file 2. [file 12866_2022_2704_MOESM2_ESM.docx]

**Supplementary tables**

**Table S1.** Diet specification for minipigs study produced by Special Diets Service (Witham, United Kingdom). Items are listed as %.

| Ingredient | Control - SDS | CDAHFD |
| --- | --- | --- |
| Cholesterol | 0 | 1 |
| Cholic acid | 0 | 0.35 |
| Fat | 2.13 | 30 (milk fat and cocoa butter |
| Protein | 13.03 | 20 |
| Choline | 0.9 | 0 |
| Methionine | 0.18 | 0.1 |
| Histidine | 0.33 | 0.57 |
| Fructose/Sucrose | 5.54 | 20 |

**Table S2.** List of primer sequences used for determining gene expression levels using qPCR in liver with Göttingen minipigs fed either control or choline-deficient-amino-acid-defined-high-fat-diet (CDAHFD) for 8 weeks.

| Gene | Forward | Reverse |
| --- | --- | --- |
| *TBP* (ref.) | AACAGTTCAGTAGTTATGAGCCAGA | AGATGTTCTCAAACGCTTCG |
| *IRS1* | GACGGCGGTTTCATCTCTTC | CCCCATGCAGATGTAGTTGC |
| *IRS2* | GACCCTGGCTTTATGTCCCT | GGAGTCTCGGCGATGGATT |
| *RHEB* | GCAGAATCTTGGAATGCAGCTT | CAGGACGACTTGCCTTGTGA |
| *MTOR* | CGCACAAGGACGGATTCCTA | CACTGTGGTCCCCGTTTTCT |
| *GCGR* | AGGGAGGTGGCTGAGATGTA | CAGGTTCGCGTGGATGTAGT |

**Table S3.** List with limit of detection values for the measurable serum amino acids.

| **Amino Acid** | **LOD** |
| --- | --- |
| Alanine | 0.133 |
| Proline | 0.019 |
| Glycine | 0.034 |
| Serine | 0.003 |
| Threonine | 0.01 |
| Aspartic acid- | 0.005 |
| Glutamic acid | 0.748 |
| Phenylalanine | 0.003 |
| Asparagine | 0.388 |
| Glutamine | 1.392 |
| Ornithine | 0.015 |
| Lysine | 0,002 |
| Tyrosine | 0,027 |
| Tryptophan | 0,012 |

**Table S4.** List of bacterial differences at family and genus levels between Göttingen minipigs fed either control or choline-deficient-amino-acid-defined-high-fat-diet (CDAHFD) for 8 weeks. DAtest statistics using DA.lli function, fdr. adjusted p-values<0.05 are listed.

| **Bacterial family** | **Bacterial genus** | **Upregulated in** | **Adjusted p-value** |
| --- | --- | --- | --- |
| *Ruminococcaceae* | *Eubacterium8"* | Control | 1.748964*10^-09^ |
| AC160630 | PAC002374 | Control | 1.168011*10^-04^ |
| *Christensenellaceae* | AY442821 | CDAHFD | 4.273404*10^-02^ |
| *Bifidobacteriaceae* | *Bifidobacterium* | CDAHFD | 4.502920*10^-03^ |
| *Muribaculaceae* | PAC001068" | CDAHFD | 2.469433*10^-02^ |
| *Christensenellaceae* | PAC001207 | CDAHFD | 1.588835*10^-02^ |
| *Ruminococcaceae* | *Phocea"* | Control | 1.185663*10^-02^ |
| *Lachnospiraceae* | *Lachnospira"* | Control | 4.287443*10^-02^ |
| *Lachnospiraceae* | *Epulopiscium"* | CDAHFD | 6.641150*10^-03^ |
| *Christensenellaceae* | PAC001360" | Control | 7.725591*10^-06^ |
| *Ruminococcaceae* | *Subdoligranulum* | Control | 7.227731*10^-03^ |
| *Ruminococcaceae* | *Ruminococcus* | Control | 5.265010*10^-03^ |
| *Lachnospiraceae* | *Lachnospira* | Control | 4.774855*10^-07^ |
| *Enterobacteriaceae* | *Escherichia"* | CDAHFD | 1.339951*10^-03^ |
| *Ruminococcaceae* | *Faecalibacterium* | Control | 2.335801*10^-06^ |
| *Lachnospiraceae* | AB506217 | Control | 9.240726*10^-03^ |
| *Ruminococcaceae* | *Monoglobus* | Control | 4.643490*10^-09^ |
| PAC001057 | PAC001108" | Control | 1.003530*10^-03^ |
| PAC001057 | PAC001108 | Control | 8.095675*10^-09^ |
| *Peptostreptococcaceae* | *Clostridioides* | CDAHFD | 2.174200*10^-02^ |
| *Ruminococcaceae* | PAC000748 | Control | 1.319323*10^-09^ |
| *Ruminococcaceae* | *Pseudoflavonifractor"* | CDAHFD | 2.540749*10^-02^ |
| *Ruminococcaceae* | *Eubacterium8* | Control | 8.953448*10^-04^ |
| *Lachnospiraceae* | *Agathobacter* | Control | 4.287443*10^-02^ |
| *Ruminococcaceae* | *Pseudoflavonifractor* | CDAHFD | 3.633453*10^-02^ |
| **Bacterial family** | **Bacterial genus** | **Upregulated in** | **p-value** |
| AC160630 | PAC001134" | Control | 1.674272*10^-07^ |
| *Streptococcaceae* | *Streptococcus"* | Control | 3.118697*10^-02^ |
| *Ruminococcaceae* | *Oscillibacter"* | CDAHFD | 2.525700*10^-04^ |
| *Lactobacillaceae* | *Lactobacillus* | CDAHFD | 3.401358*10^-02^ |
| *Ruminococcaceae* | *PAC000672"* | Control | 6.653307*10^-05^ |
| *Lachnospiraceae* | *Agathobacter"* | Control | 1.095551*10^-03^ |
| *Ruminococcaceae* | *Ruminococcus* | Control | 3.848433*10^-02^ |
| *Lachnospiraceae* | *Ruminococcus4* | CDAHFD | 1.416035*10^-03^ |
| *Lachnospiraceae* | *FMWZ* | Control | 4.273404*10^-02^ |
| *Clostridiaceae* | *Clostridium"* | CDAHFD | 4.989096*10^-02^ |
| *Lachnospiraceae* | *Eubacterium24"* | Control | 3.633453*10^-02^ |
| AC160630 | AB494828" | Control | 9.165353*10^-10^ |
| *Ruminococcaceae* | *Sporobacter"* | CDAHFD | 5.282669*10^-03^ |
| *Ruminococcaceae* | AB218327 | Control | 1.753259*10^-02^ |
| AC160630 | HQ716269 | Control | 1.168011*10^-04^ |
| *Bacteroidaceae* | *Bacteroides"* | CDAHFD | 8.923251*10^-03^ |
| *Ruminococcaceae* | *Eubacterium23* | CDAHFD | 3.940653*10^-03^ |
| AC160630 | AB494828 | Control | 4.955680*10^-10^ |
| *Muribaculaceae* | PAC001112 | Control | 8.039471*10^-10^ |
| *Desulfovibrionaceae* | *Desulfovibrio* | CDAHFD | 2.722370*10^-04^ |
| *Muribaculaceae* | PAC001112" | Control | 1.500975*10^-02^ |
| *Ruminococcaceae* | *Sporobacter* | CDAHFD | 4.287443*10^-02^ |
| *Ruminococcaceae* | *Oscillibacte* | CDAHFD | 1.753259*10^-02^ |

**Table S5.** Results of multiple linear mixed regression to predict glucagon level having imidazole propionate and total amino acid as fixed variables. Stepwise backwards regression was used to construct the model.

| *Independent variables* | *Estimates* | *Std. Error* | *95% CI* | *P-value* |
| --- | --- | --- | --- | --- |
| Imidazole propionate | 0.025 | 0.0071 | (0.01 ; 0.04) | 0.0068 |
| Total amino acid | 0.20 | 0.13 | (-0.06 ; 0.47) | 0.16 |

F-statistics: (final): F(1,10) = 10.93; p = 0.0079; Adj. *R^2^* = 0.47

**Table S6.** Results from multiple linear regression predicting hepatic fibrosis having members of the *Ruminococcus* genus and total bile acids as independent variable. Stepwise backward regression was used to construct the model.

| *Independent variables* | *Estimates* | *Std. Error* | *9% CI* | *P-value* |
| --- | --- | --- | --- | --- |
| *Ruminococcus* genus | -0.64 | 0.23 | (-1.09 ; -0.19) | 0.021 |
| Total bile acid | 0.026 | 0.011 | (0.001 ; 0.05) | 0.042 |

F-statistics: (final): F(2,9) = 8.57; p = 0.0083; Adj. *R^2^* = 0.58
