# Supplementary material for: Marked gut microbiota dysbiosis and increased imidazole propionate are associated with a NASH Göttingen Minipig model
Source: BMC Microbiol. 2022 Dec 1;22:287. doi: 10.1186/s12866-022-02704-w (PMC9717514; doi:10.1186/s12866-022-02704-w)
Supplement: Supplementary file 3 — Additional file 3. [file 12866_2022_2704_MOESM3_ESM.docx]

**Supplementary methods**

**DNA extraction, sequencing and pre-processing of raw data**

Fecal samples were thawed at 4°C, re-suspended in ultrapure water (1:2 feces/water) and homogenized in filter bags for 1 min at high speed (Lab Seward, BA7021). 1.5 ml of the fecal slurry was centrifuged at 13,000×g for 10 min at room temperature. An amount of ~200 mg of the fecal pellet from each individual sample was used for DNA extraction using the Bead-Beat Micro AX Gravity kit (A&A Biotechnology, Gdynia, Poland) following the instructions of the manufacturer. Gut prokaryotic composition was determined using NexSeq 500 (Illumina, CA, USA) based 16S rRNA gene-amplicon sequencing by targeting the V3 region with paired primers designed with adapters for the Nextera Index Kit® (Illumina, CA, USA): NXt_338_F: 5’- TCG TCG GCA GCG TCA GAT GTG TAT AAG AGA CAG ACW CCT ACG GGW GGC AGC AG -3’ and NXt_518_R: 5’- GTC TCG TGG GCT CGG AGA TGT GTA TAA GAG ACA GAT TAC CGC GGC TGC TGG -3’. The library preparation was accomplished by 2-steps PCR works. The amplification profile (1st PCR), barcoding (2nd PCR), amplicon library purification and sequencing were performed as previously described by Pyndt Jørgensen et al^1^.

**Analysis of high-throughput amplicon sequencing**

The raw dataset containing pair-ended reads with corresponding quality scores were merged and trimmed using the following settings, -fastq_minovlen 100, -fastq_maxee 2.0, -fastq_truncal 4, -fastq_minlen 130. Finding unique reads and deconvoluting from chimeric reads and constructing high quality (97% similarity level) Operational Taxonomic Units (OTUs) was conducted using the UPARSE pipeline ^2^2013 coupled to the GreenGenes 16S rRNA gene collection as a reference database^3^. The sequencing depth was on average 68194 read per sample before filtering going down to 62426 after filtering. The raw reads were filtered and low-abundance OTUs across all samples with below 0.005% were removed using R, an open-source statistical software V 4.0.3. The differences in sequencing depth were corrected using Cumulative Sum Scaling (CSS) normalization *via* MetagenomeSeq 1.32.0^4^.

**Gene expression**

50 mg of snap-frozen liver tissue were used for RNA isolation with the RNeasy Mini kit including DNAse treatment (Qiagen, Hilden, Germany) as described in (23). Concentration and purity of the RNA samples were analyzed by Nanodrop ND-1000 spectrophotometer (NanoDrop technologies, Wilmington, USA). RNA integrity was assessed firstly by visual inspection in agarose gel and subsequently, by a RNA-quality index (RQI) score in an Experion machine using the RNA stdSens kit (BioRad, Denmark). All samples had a RQI between 8.9-10 (average= 9.4) and were all accepted for further processing.

Subsequently, cDNA synthesis was done in duplicate for each RNA sample as described in ^5^. Briefly, 0.5 µgr of liver RNA-DNase treated was mixed with 0.5 μl Improm-II reverse transcriptase (Promega), 0.25 μg 1:3 OligodT/Random primers, 2 μl ImProm-II buffer, 10 units RNasin Ribonuclease inhibitor (Promega), 2.5 mM MgCl2 and 2 mM dNTP in a total volume of 10 μl. Incubation was done following manufacturer’s recommendations. Two negative controls without reverse transcriptase were done in parallel. The cDNA samples were diluted 16 times prior to quantitative real-time PCR (qPCR) and stored at -80 °C until use.

QPCR was performed in a CFX96^TM^ Real-Time System (Bio-Rad) mixing 5µl SsoAdvanced Universal SYBR® green supermix (Bio-Rad), 0.08 µl forward and reverse primer pool 50µM (Sigma-Aldrich, sequences are listed in supplementary table S2), 3.92 µl nuclease free water and 1 µl of 1:16 diluted cDNA in a total volume of 10 µl. PCR cycling condition were: 30 seconds 95°C followed by 40 cycles of 10 seconds denaturation at 95°C and 30 seconds 60°C annealing/extension ending with a melting curve

Investigated genes were selected on the basis of relevance for the tissue and the pathways of interest for the present study (imidazole propionate activation pathways and genes related to the hormone glucagon). Primers (Sigma-Aldrich) were designed using primer 3 (http://bioinfo.ut.ee/primer3/) or primer blast (https://www.ncbi.nlm.nih.gov/tools/primer-blast/) with standard settings. They were designed, if possible, to span over an intron and yield ampliqons of 74-200 nucleotides (supplementary Table S2). Primers were validated prior to analysis using a dilution row of a pool of cDNA of all the samples included in the analysis in order to assess levels of amplification < 40 cycles and PCR efficiency between 80% and 120%. Melting curve analysis was included to assess the specificity of each assay. Genex 6 (MultiD Analyses AB, Gothenburg, Sweden) was used to process the raw qPCR data. Reference gene were determined by using geNorm and NormFinder algorithms in Genex6 and based on previous studies^6, 7^. Briefly, qPCR data was corrected by PCR efficiency for each assay, normalized to Tata-Binding Protein (TBP) gene, qPCR replicates were averaged and relative quantities in respect to the lowest expressed sample in each assay were calculated. Finally, data were log2 transformed before being analyzed using Student´s t-test including fdr adjustment for multiple testing by Benjamin, Krieger, and Yekutieli using PRISM v9.0.1.

**Serum and faecal metabolite analysis**

All serum and faecal samples were randomized prior to the sample preparation. Aliquots of 30 µL blood serum were subjected for protein precipitation using 400µL methanol, containing internal standards purchased from Sigma-Aldrich, Germany (1 µg/mL deuterium-labeled succinic acid, deuterium-labeled glutamic acid, deuterium-labeled valine and 5 µg/mL heptadecanoic acid). After vortex mixing, incubating on ice for 30 min and centrifugation at 9400 x g for 3 min, 350 µL of supernatants were collected. The supernatants were then evaporated under gentle nitrogen flow to dryness after which a two-step derivatization was performed. First, 25 µL of 20 mg/mL MOX reagent in pyridine was added and samples were incubated at 45 °C for 60 minutes. Secondly, 25 µL MSTFA was added and samples were incubated once again for 60 min at 45 °C. Retention index mixture (10µg/mL n-alkanes) was added to each sample before the analysis on Agilent 7890B gas chromatograph (GC) coupled to 7200 triple quad time of flight mass spectrometer (Q-TOF/MS) instrument. Initial helium flow was set to 1.2 mL/min, increasing to 2.4 mL/min after 16 minutes. Oven temperature program was kept at 50 °C for 5 minutes, with 20 °C/min increase to 270 °C, and then 40 °C/min to final temperature 300 °C (5 min). Samples with injection volume of 1 µL and 100:1 split ratio were injected using PTV injector set to 70 °C, heated to 300 °C at 120 °C/min. Zebron ZB-SemiVolatiles column (20m length, 0.18mm inner diameter, 0.18µm film thickness) purchased from Phenomenex Inc., USA was used to achieve a chromatographic separation. EI source was set to 250 °C, 70 eV and 35 µA emission with 3 minutes solvent delay. Quadrupole was kept at 150 °C having 1.5 mL/min N_2_ collision gas flow. The data was acquired at 55-650 amu mass range and 200 ms/spectrum acquisition time.

Six-points calibration curves at 0.1-80 µg/mL range 2-hydroxybutyric acid, 3-hydroxybenzoic acid, 3-hydroxybutyric acid, 5-hydroxyindole-3-acetic acid, alanine, arachidonic acid, ascorbic acid, asparagine, aspartic acid, cholesterol, citric acid, decanoic acid, fructose, fructose-6-phosphate, fumaric acid, glucose-6-phosphate, glutamic acid, glutamine, glyceraldehyde, glyceraldehyde-3-phosphate, glycerol-3-phosphate, glycine, indole-3-acetic acid, indole-3-lactic acid, indole-3-propionic acid, isocitric acid, isoleucine, lactic acid, leucine, linoleic acid, lysine, malic acid, methionine, octanoic acid, oleic acid, ornithine, palmitic acid, phenylalanine, phosphoenolpyruvic acid, proline, ribose-5-phosphate, serine, stearic acid, succinic acid, threonine, tryptophan, tyrosine and valine from Sigma-Aldrich, Germany.

SCFAs extraction in faecal material was processed by adding 1 mL of 5 mM aqueous NaOH containing internal standard (5 µg/mL hexanoic acid-d_4_ and 5 µg/mL valine-d_8_) to 100 mg aliquots. Sample was homogenized with a micropestle and mixed for 10 min at a shaker (300 rpm). After shaking, the sample was centrifuged for 20 min at 13200 x g at 4°C. 300µl of MQ water, 500µL propanol/pyridine mixture solvent (v/v = 3:2), 100 µL of propyl-chloroformate were added to 500 µL of faecal water obtained after centrifugation. The sample was vortexed and ultrasonicated for 1 min. After adding 300 µL of hexane, the sample was vortexed and centrifuged for 5 min at 2000 x g. 300 µL from the hexane layer was collected in a glass vial. 200µL of retention index standards in hexane were added before analysis. Another aliquot of 100 mg faecal sample for dry weights determination were freeze-dried overnight at -50°C.

Acquisition of BCFAs was done using an Agilent 7890A GC to an Agilent 5975C MS equipped with an electron ionization (EI) source (230 °C). GC separation was achieved using a DB-5MS capillary column, 30 m × 0.25 mm i.d. × 0.25 μm film thickness (Agilent Technologies, Atlanta, GA, USA). The oven temperature was as follows: 45 °C (4 min); 10 °C/min to 70 °C; 3 °C/min to 85 °C; 5°C/min to 110 °C; 30 °C/min to 300 °C (5 min). 1 µL of samples were injected in splitl mode (split ratio 5:1) and carried out by carrier gas (helium) at 260 ℃ with a constant flow of 1.0 mL/min. The data was acquired in scan mode and mass range was between 50 and 300 amu.

**Reference list**

1. Pyndt Jørgensen B, Hansen JT, Krych L, Larsen C, Klein AB, Nielsen DS, et al. A possible link between food and mood: dietary impact on gut microbiota and behavior in BALB/c mice. PLoS One 2014; 9:e103398.

2. Edgar RC. UPARSE: highly accurate OTU sequences from microbial amplicon reads. Nat Methods 2013; 10:996-8.

3. McDonald D, Price MN, Goodrich J, Nawrocki EP, DeSantis TZ, Probst A, et al. An improved Greengenes taxonomy with explicit ranks for ecological and evolutionary analyses of bacteria and archaea. Isme j 2012; 6:610-8.

4. Paulson JN PMaBH. metagenomeSeq: Statistical analysis for sparse high-throughput sequncing. <http://www.cbcb.umd.edu/software/metagenomeSeq>, 2013.

5. Pedersen HD, Galsgaard ED, Christoffersen B, Cirera S, Holst D, Fredholm M, et al. NASH-inducing Diets in Göttingen Minipigs. J Clin Exp Hepatol 2020; 10:211-21.

6. Nygard AB, Jørgensen CB, Cirera S, Fredholm M. Selection of reference genes for gene expression studies in pig tissues using SYBR green qPCR. BMC Mol Biol 2007; 8:67.

7. Cirera S, Taşöz E, Juul Jacobsen M, Schumacher-Petersen C, Østergaard Christoffersen B, Kaae Kirk R, et al. The expression signatures in liver and adipose tissue from obese Göttingen Minipigs reveal a predisposition for healthy fat accumulation. Nutr Diabetes 2020; 10:9.
